# Role of Prolactin in the Recovered T-Cell Development of Early Partially Decapitated Chicken Embryo

**DOI:** 10.1155/1998/93086

**Published:** 1998

**Authors:** J. Moreno, A. Varas, A. Vicente, A. G. Zapata

**Affiliations:** 1 National Center of Microbiology Institute of Salud Carlos III 28220 Majadahonda Madrid Spain; 2 Department of Cell Biology Faculty of Medicine Complutense University Madrid 28040 Spain; 3 Department of Cell Biology Faculty of Biology Complutense University Madrid 28040 Spain

**Keywords:** Chicken thymus, prolactin, T-cell development

## Abstract

Although different experimental approaches have suggested certain regulation of the mammalian immune system by the neuroendocrine system, the precise factors involved in the process are largely unknown. In previous reports, we demonstrated important changes in the thymic development of chickens deprived of the major neuroendocrine centers by the removal of embryonic prosencephalon at 33-38 hr of incubation (DCx embryos) (Herradón et al., 1991; Moreno et al., 1995). In these embryos, there was a stopping of T-cell maturation that resulted in an accumulation of the most immature T-cell subsets (CD4^-^CD8^-^ cells and CD4^-^CD8^1o^ cells) and, accordingly, in decreased numbers of DP (CD4^+^CD8^+^) thymocytes and mature CD3^+^TcR*αβ* ^+^ cells, but not CD3^+^TcR*γδ* lymphocytes. In the present work, we restore the thymic histology as well as the percentage of distinct T-cell subsets of DCx embryos by supplying recombinant chicken prolactin, grafting of embryonic pituitary gland, or making cephalic chick-quail chimeras. The recovery was not, however, whole and the percentage of CD3^+^TcR*αβ* thymocytes did not reach the normal values observed in 17-day-old control Sham-DCx embryos. The results are discussed on the basis of a key role for prolactin in chicken T-cell maturation. This hormone could regulate the transition of DN (CD4^-^CD8^-^) thymocytes to the DP (CD4^+^CD8^+^) cell compartment through its capacity for inducing IL-2 receptor expression on the former.


*Developmental Immunology*, 1998, Vol. 5, pp. 183–195
Reprints available directly from the publisher
Photocopying permitted by license only

© 1998 OPA (Overseas Publishers Association)
Amsterdam B.V. Published under license
under the Harwood Academic Publishers imprint,
part of the Gordon and Breach Publishing Group
Printed in Malaysia

### Role of Prolactin in the Recovered T-Cell Development of Early Partially Decapitated Chicken Embryo

J. MORENO[a], A. VARAS[c], A. VICENTE[b] and A. G. ZAPATA[c*]

[a]*National Center of Microbiology, Institute of Salud Carlos III, 28220 Majadahonda (Madrid), Spain*; [b]*Department of Cell Biology, Faculty of Medicine, Complutense University, 28040 Madrid, Spain*; [c]*Department of Cell Biology, Faculty of Biology, Complutense University, 28040 Madrid, Spain*

(*Received 6 March 1997; In final form 6 May 1997*)

Although different experimental approaches have suggested certain regulation of the mammalian immune system by the neuroendocrine system, the precise factors involved in the process are largely unknown. In previous reports, we demonstrated important changes in the thymic development of chickens deprived of the major neuroendocrine centers by the removal of embryonic prosencephalon at 33-38 hr of incubation (DCx embryos) (Herradón et al., 1991; Moreno et al., 1995). In these embryos, there was a stopping of T-cell maturation that resulted in an accumulation of the most immature T-cell subsets ($CD4^-CD8^-$ cells and $CD4^-CD8^{lo}$ cells) and, accordingly, in decreased numbers of DP ($CD4^+CD8^+$) thymocytes and mature $CD3^+TcR\alpha\beta^+$ cells, but not $CD3^+TcR\gamma\delta$ lymphocytes. In the present work, we restore the thymic histology as well as the percentage of distinct T-cell subsets of DCx embryos by supplying recombinant chicken prolactin, grafting of embryonic pituitary gland, or making cephalic chick-quail chimeras. The recovery was not, however, whole and the percentage of $CD3^+TcR\alpha\beta^+$ thymocytes did not reach the normal values observed in 17-day-old control Sham-DCx embryos. The results are discussed on the basis of a key role for prolactin in chicken T-cell maturation. This hormone could regulate the transition of DN ($CD4^-CD8^-$) thymocytes to the DP ($CD4^+CD8^+$) cell compartment through its capacity for inducing IL-2 receptor expression on the former.

*Keywords:* Chicken thymus, prolactin, T-cell development

### INTRODUCTION

Numerous studies have emphasized the importance of the pituitary gland for the development and maturation of mammalian lymphoid organs, mainly thymus, although the factors involved remain to be conclusively characterized. Snell-Bagg and Ames dwarf strain mice, which do not contain acidophilic cells in the pituitary gland resulting in a deficient production of growth hormone (GH), prolactin (PRL), and other neuroendocrine mediators, show early thymic involution (Baroni, 1967; Baroni et al., 1967; Duquesnoy,

*Corresponding author, phone 34-1-394 49 79, FAX 34-1-394 49 81, E-mail Zapata@eucmax.sim.ucm.es

1972) with decreased numbers of DP ($CD4^+CD8^+$) cells (Cross et al., 1992; Murphy et al., 1992a, 1992b). Moreover, grafting of syngeneic pituitary glands or administration of PRL and/or GH generally totally or partially restores the immune system (Berczi et al., 1981; Fabris et al., 1989; Murphy et al., 1992a; Esquifino et al., 1991), but results are frequently contradictory. In fact, obvious difficulties for *in vivo* manipulation of mammalian fetuses make it difficult to obtain direct evidence on the mechanisms that govern the establishment of such neuroendocrine immune relationships during ontogeny.

In chickens, an excellent experimental model for ontogenetical studies, we reported, several years ago, important morphological changes in the thymus of embryos deprived of the major neuroendocrine centers including the pineal system, hypothalamus, and pituitary gland, by early partial decapitation (DCx embryos) (Herradón et al., 1991), confirming previous data by Jankovic et al. (1978, 1981, 1982). More recently, we have demonstrated that such changes affect differently the distinct T-cell subsets, resulting in an accumulation of the most immature elements including DN ($CD4^-CD8^-$) cells and $CD8^{lo}CD4^-$ cells, and an almost total disappearance of DP ($CD4^+CD8^+$) cells and TcR$\alpha\beta$-expressing cells with few modifications in the pattern of $\gamma\delta$ T-cell differentiation (Moreno et al., 1995).

Although Jankovic and co-workers attributed the delayed development of the immune system of DCx embryos to the profound disturbance of their neuroendocrine system, the factor(s) involved were not identified and they did not carry out any substitutive approach to recover it. In the present study, we have used different experimental approaches to recover the development of T-cell subsets of DCx chicken embryos. Recombinant chicken PRL administered onto the chorioallantoid membrane of DCx embryos (DCx+PRL embryos) restored T-cell development considerably but not totally. Pituitary glands from 11-day old embryonic chickens grafted onto the chorioallantoid membrane of 11-day old DCx embryos (DCx+Hyp embryos) also induced significant, but not total, recovery of the thymic T-cell subsets, whereas cephalic chick-quail chimeras show an almost complete recovery of the chicken T-cell system. Taken together, these results support a role for PRL in the maturation of T-cell precursors during chicken ontogeny.

### RESULTS

The thymus of DCx embryos showed a decreased size and delayed development with a high number of large, blast cells, enlarged connective tissue trabeculae, and absence of a clear cortico-medullary demarcation (Figure 1B). The *in situ* immunohistochemical study, however, did not demonstrate important changes in the expression of various cell markers specific for thymic epithelial cells, macrophages, or interdigitating cells, although a careful morphometrical analysis was not carried out (for details, see Moreno et al., 1995). On the other hand, the thymus of DCx+PRL (Figure 1C), DCx+Hyp embryos (Figure 1D), and chimeras (Figure 1E) showed, in general, a normal size and morphology although the two first ones exhibited a slight increase of the medullary area and enlarged trabeculae (Figures 1C and 1D) when compared to the Sham-DCx embryos (Figure 1A).

According to the expression of either cell-surface molecule recognized by the mAb CVI-His-$C_7$, the three experimental approaches used recovered the thymic T cells of DCx embryos. Although again the values recorded in both DCx+PRL and DCx+Hyp embryos did not reach those of control, Sham-DCx embryos (Table II). On the contrary, values in chimeric embryos were the same as control values except for the proportion of CD28 positive cells found at day 15 of incubation. Accordingly, all thymocytes, including those of the subcapsulary cortex, which in DCx embryos appeared unstained (Figure 2A), were positive for the antigenic determinant recognized by mAb CVI-His-$C_7$ (Figure 2B). Similar results were obtained using a battery of mAbs MUI-83, CT1, and 5-5, which seems to recognize immature thymocytes (Table II). The proportion of positive cells of chimeras was similar to that of control, Sham-DCx embryos, except for CT1 positive cells at day 17 of incubation, whereas in both DCx+PRL and DCx+Hyp embryos, although the values were always higher than those found in DCx embryos, they did not reach control numbers (Table II).

The percentage of immature T cells, including DN ($CD4^-CD8^-$) cells and $CD8^{lo}CD4^-$ cells, decreased significantly at days 15 and 17 of incubation in the three experimental groups of DCx embryos used compared to nontreated DCx embryos, but it did not reach the control values found in 17-day-old Sham-DCx embryos (Table III). Accordingly, the numbers of DP ($CD4^+CD8^+$) cells increased, without reaching control values, in DCx+PRL and DCx+Hyp embryos and chimeras (Table III). In contrast, although there was a small increase in the proportion of mature $CD4^-CD8^{hi}$ cells compared to the values observed in DCx embryos, differences were not statistically significant (Table III). The CD4 cell marker was always co-expressed with CD8, and the $CD4^+CD8^-$ T-cell population was irrelevant in all groups analyzed (Table III).

The immunostaining of thymic sections with mAb specific either to CD4 or CD8 confirmed the cytofluorometric results. In the three experimental groups, the pattern of staining was similar and resembled that found in control (Figure 3A). Both CD4- and CD8-expressing cells occupied mainly all the thymic cortex, including the subcapsulary and outer area (Figures 3C, to 3F), which in DCx embryos appeared unstained with a few positive cells in the medulla (Figure 3B).

On the other hand, although the percentage of TcR$\alpha\beta$-expressing cells increased in DCx+PRL and DCx+Hyp embryos, and chimeras, compared to the values observed in 17-day-old DCx embryos, it was still lower than control numbers (Table IV). In contrast, no significant differences were observed in

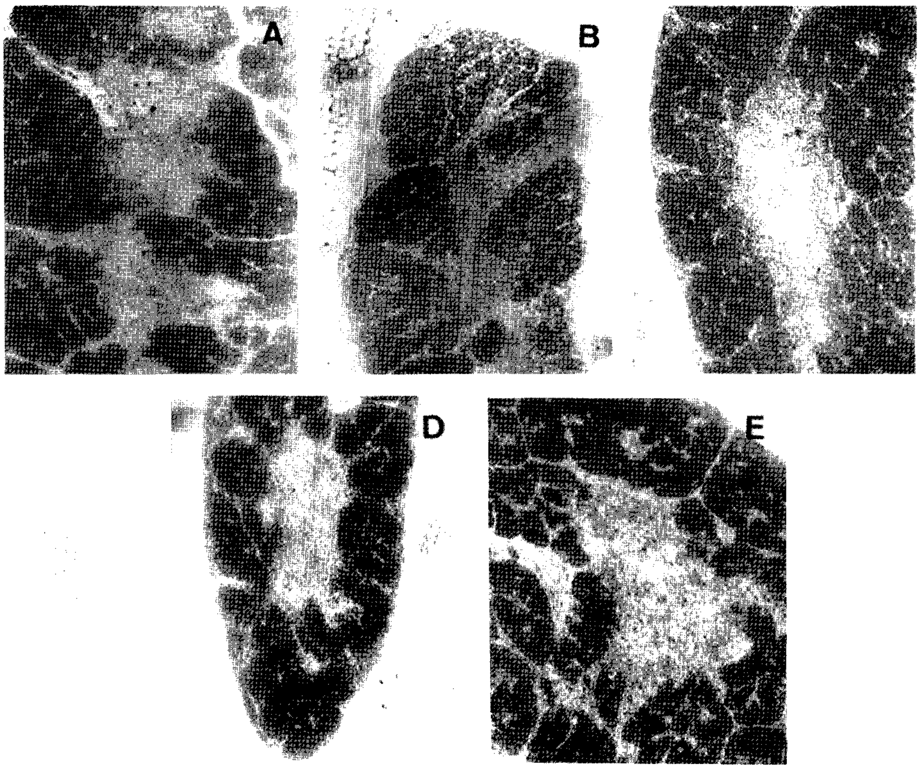

FIGURE 1 Histological sections of thymic lobes from 17-day-old (A) Sham-DCx, (B) DCx, (C) DCx+PRL, (D) DCx+Hyp, and (E) chimera embryos were stained according to methylene blue. Note the morphological similarities among thymi from (A) control, Sham-DCx, and (C, D, and E), recovered embryos and the scarce development of (B) DCx-thymus. Magnification: × 50.

TABLE I Monoclonal Antibodies used in this Study.

| mAbs | Specificity | Source |
|---|---|---|
| CVI-His-$C_7$ | Pan-leucocytes | S.H.M. Jeurissen, Lelystad, Holland |
| 2-4 | CD28 | O. Vainio, University of Turku, Finland |
| 5-5 | Immature thymocytes | O. Vainio, University of Turku, Finland |
| MUI-83 | Immature thymocytes | R.L. Boyd, Monash University, Australia |
| CT1 | Immature thymocytes | M.D. Cooper, University of Alabama, USA |
| CT3 | CD3 | M.D. Cooper, University of Alabama, USA |
| CT4 | CD4 | M.D. Cooper, University of Alabama, USA |
| CT8 | CD8 | M.D. Cooper, University of Alabama, USA |
| TCR1 | TcR$\alpha\beta$ | M.D. Cooper, University of Alabama, USA |
| TCR2 | TcR$\gamma\delta$ | M.D. Cooper, University of Alabama, USA |

TABLE II Proportion of CVI-His C7, 2-4, 5-5, CT1, and MUI-83 Positive Cells in Sham-DCx, DCx, DCx+PRL, DCx+Hyp embryos and Chimeras[a]

| | Day of incubation | CVI-His-$C_7$ | 2-4 (CD28) | 5-5 | CT1 | MUI-83 |
|---|---|---|---|---|---|---|
| Sham-DCx | 15 | 84.3 ± 10.4 | 91.3 ± 1.5 | 60.0 ± 5.4 | 76.1 ± 4.0 | 70.1 ± 5.6 |
| | 17 | 93.0 ± 1.6 | 90.8 ± 1.0 | 68.6 ± 5.1 | 88.7 ± 2.9 | 80.7 ± 3.1 |
| DCx | 15 | 67.7 ± 4.2* | 72.1 ± 3.8** | 49.7 ± 12.3 | 59.3 ± 4.6** | 47.4 ± 11.2** |
| | 17 | 51.2 ± 14.7** | 58.0 ± 5.6** | 34.2 ± 7.8** | 29.8 ± 10.2** | 29.8 ± 12.7* |
| DCx+PRL | 15 | ND | 90.1 ± 1.4 | ND | 85.6 ± 2.1* | ND |
| | 17 | 86.5 ± 0.3** | 84.8 ± 4.3* | 54.4 ± 7.8* | 68.5 ± 6.1** | 58.3 ± 5.6** |
| DCx+Hyp | 15 | 70.2 ± 7.6 | 77.7 ± 1.5** | 52.9 ± 4.9 | 49.9 ± 6.0** | 56.2 ± 8.9* |
| | 17 | 86.7 ± 6.4* | 88.7 ± 3.9 | 59.4 ± 3.1* | 69.3 ± 7.5** | 48.8 ± 11.8** |
| Chimera | 15 | 87.9 ± 5.6 | 86.4 ± 3.4* | 75.5 ± 8.2* | 61.4 ± 9.1* | 76.7 ± 7.6 |
| | 17 | 92.6 ± 4.2 | 91.9 ± 2.0 | 76.0 ± 8.4 | 81.2 ± 3.8* | 77.6 ± 3.8 |

[a]$p \leq 0.1$ and $p \leq 0.05$ significant differences to control, Sham-DCx values are marked as * and **, respectively.

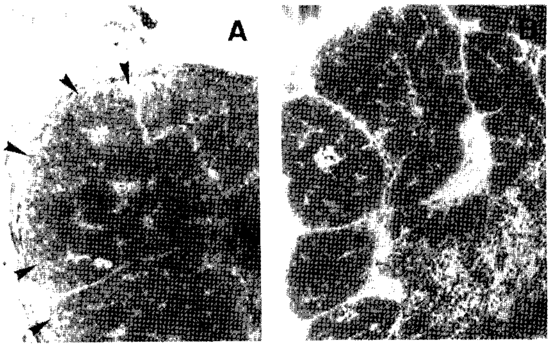

FIGURE 2 Thymi of 17-old-day (A) DCx and (B) chimera embryos stained with the mAb CVI-His-$C_7$. Note the lack of staining in the subcapsulary cortex of DCx embryo (arrowheads). Magnification: × 62.

the proportion of $\gamma\delta$ positive T cells, although the variations between each of the embryos analyzed were considerably high (Table IV). In all groups of embryos, the highest values occurred on day 15, decreasing thereafter (Table IV). In parallel, the recovery of $CD3^+$ T-cell subsets observed in the DCx+PRL and DCx+Hyp embryos as well as in the chimeras was also incomplete and reflected the expression of these cell-surface molecules in TcR$\gamma\delta$-expressing cells (Table IV).

### Endocrinological Background

Like the DCx embryos (Figures 4A to 4D), DCx+PRL embryos showed considerable delay in the develop-

TABLE III Percentages of DN (CD4$^-$CD8$^-$), CD4$^-$/CD8$^{lo}$, CD4$^-$/CD8$^{high}$, DP (CD4$^+$CD8$^+$), and CD4$^+$CD8$^-$ Cells in Sham-DCx, DCx, DCx+PRL, DCx+Hyp Embryos and Chimeras[a]

| | Day of incubation | CD4−/8− | CD4−/8low | CD4−/8high | CD4+/8+ | CD4+/8− |
|---|---|---|---|---|---|---|
| Sham-DCx | 15 | 43.8 ± 6.6 | 13.0 ± 1.6 | 17.9 ± 3.5 | 24.0 ± 4.2 | 1.0 ± 0.4 |
| | 17 | 17.5 ± 1.4 | 7.0 ± 1.0 | 12.2 ± 2.0 | 64.1 ± 2.7 | 1.0 ± 0.1 |
| DCx | 15 | 59.1 ± 14.7** | 25.3 ± 7.9** | 9.0 ± 6.4* | 6.0 ± 1.6** | 0.3 ± 0.1 |
| | 17 | 42.6 ± 4.2** | 34.1 ± 6.2** | 14.6 ± 3.0 | 8.1 ± 3.0** | 0.5 ± 0.2 |
| DCx+PRL | 15 | 33.8 ± 5.3 | 17.5 ± 0.8** | 19.6 ± 2.8 | 28.8 ± 4.1 | 0.4 ± 0.1 |
| | 17 | 23.1 ± 1.7** | 10.4 ± 1.9* | 14.0 ± 6.0 | 45.9 ± 6.6** | 1.4 ± 0.1 |
| DCx+Hyp | 15 | 42.8 ± 4.9 | 16.2 ± 3.7 | 18.1 ± 6.6 | 21.7 ± 5.6 | 1.6 ± 0.6 |
| | 17 | 26.6 ± 3.0** | 9.7 ± 0.9* | 18.7 ± 2.0** | 48.0 ± 2.8** | 1.2 ± 0.4 |
| Chimera | 15 | 38.3 ± 11.8 | 18.8 ± 4.0 | 20.7 ± 4.0* | 20.3 ± 2.9 | 1.1 ± 0.4 |
| | 17 | 27.0 ± 13.3** | 11.8 ± 0.3** | 18.6 ± 10.2 | 40.7 ± 6.9** | 1.6 ± 0.7 |

[a]$p \leq 0.1$ and $p \leq 0.05$ significant differences to control, Sham-DCx values are marked as * and **, respectively.

ment of thyroid (Figure 5A), adrenal glands (Figure 5B) and gonads (Figures 5C and 5D). In contrast, the development of these endocrine organs in both pituitary gland grafted DCx embryos (Figures 6A to 6D) and chimeras (Figures 7A to 7D) was generally similar to that exhibited by control, Sham-DCx embryos (Figures 8A to 8D), although in the first ones, they showed a slight delay.

RIA analysis revealed a lack of GH in all the five groups of chicken embryos at both 15 and 17 days of

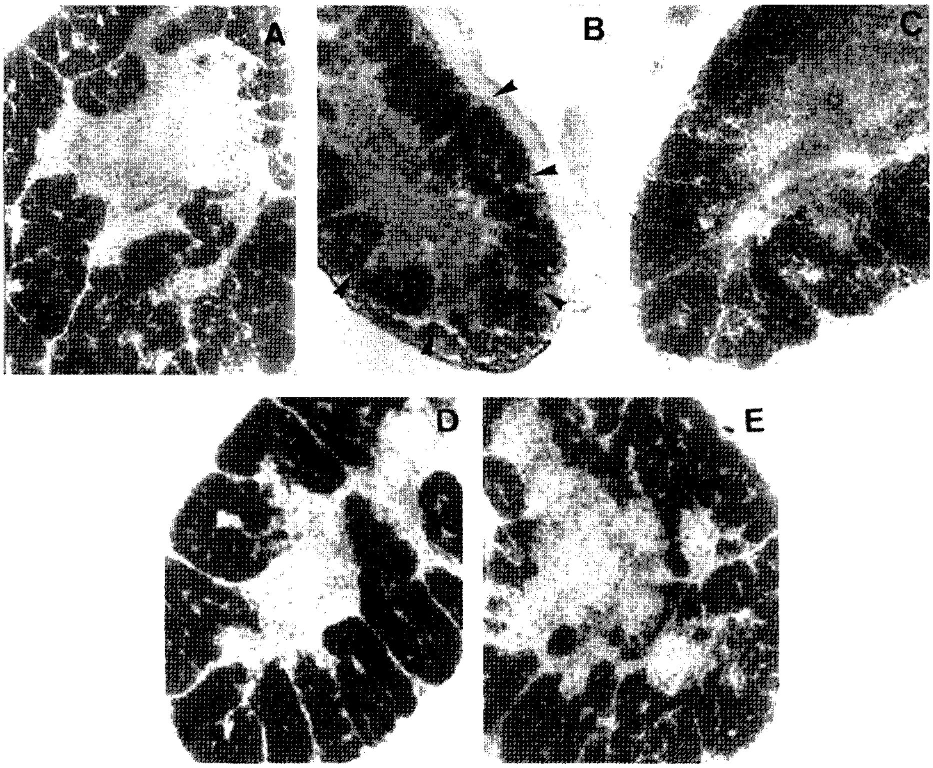

FIGURE 3 CD8-positive cells in the thymus of 17-day-old (A) Sham-DCx, (B) DCx, (C) DCx+PRL, (D) DCx+Hyp, and (E) chimera embryos. An enlarged unstained subcapsulary cortex (arrowheads) that contains CD8 negative cells appears in the (B) DCx thymus. Magnification: × 62.

TABLE IV Percentages of CD3-Positive Cells, TcR$\alpha\beta$ Cells, and TcR$\gamma\delta$ Cells in Sham-DCx, DCx+PRL, and DCx+Hyp Embryos and Chimeras[a]

| | Day of incubation | CD3 | TcR$\alpha\beta$ | TcR$\gamma\delta$ |
|---|---|---|---|---|
| Sham-DCx | 15 | 36.3 ± 4.2 | 8.4 ± 2.0 | 22.7 ± 2.3 |
| | 17 | 44.8 ± 9.2 | 21.8 ± 1.4 | 12.3 ± 2.5 |
| DCx | 15 | 22.0 ± 1.4** | 4.5 ± 0.7** | 18.3 ± 1.0** |
| | 17 | 19.0 ± 5.4** | 7.4 ± 3.2** | 15.3 ± 5.7 |
| DCx+PRL | 15 | 32.8 ± 3.1 | 5.0 ± 0.4 | 22.6 ± 2.0 |
| | 17 | 24.1 ± 1.5** | 13.7 ± 2.5** | 9.1 ± 2.5 |
| DCx+Hyp | 15 | 20.8 ± 1.2** | 2.9 ± 0.6** | 15.2 ± 3.1** |
| | 17 | 32.6 ± 12.0 | 12.7 ± 0.8** | 11.8 ± 0.9 |
| Chimera | 15 | 32.9 ± 6.9 | 3.6 ± 0.9* | 30.7 ± 3.6** |
| | 17 | 29.9 ± 2.7 | 11.4 ± 5.4** | 16.8 ± 6.4 |

[a]$p \leq 0.1$ and $p \leq 0.05$ significant differences to control, Sham-DCx values are marked as * and **, respectively.

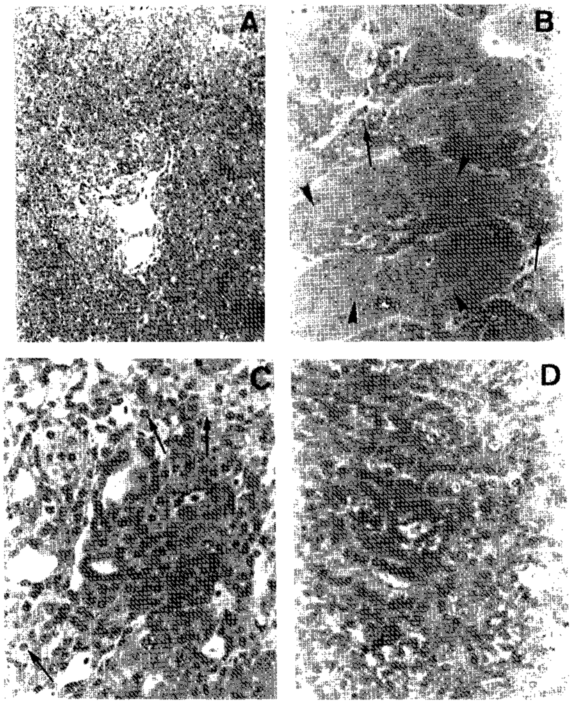

FIGURE 4 (A) Thyroid gland, (B) adrenal gland, (C) ovary, and (D) testis of a 17-day-old DCx embryo were embedded in plastic resin for light microscopy examination. Note (A) the lack of fully developed thyroid follicles devoid of colloidal fluid, the few numbers of both chromaffin cells (arrows), and interrenal cells (arrowheads) of the (B) adrenal gland, the scarcely developed intertitial cells (arrows) of the (C) ovary and (D) the lack of well-developed seminiferous tubules in the testis. Magnification: × 175.

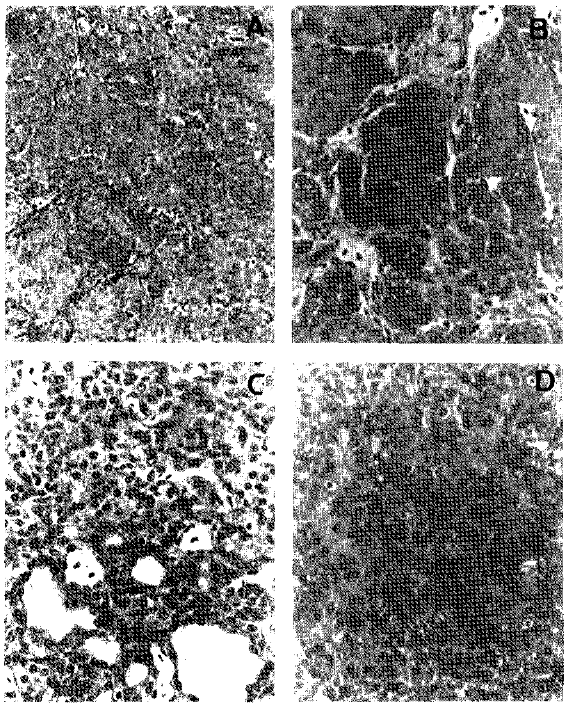

FIGURE 5 (A) Thyroid gland, (B) adrenal gland, (C) ovary, and (D) testis of a 17-day-old DCx+PRL embryo were embedded in plastic resin for light microscopy examination. The prolactin treatment does not recover the normal histology of endocrine organs, which resembles that found in the DCx embryos (Figure 4). Magnification: × 175.

incubation (data not shown). On the other hand, relative plasma levels of PRL were similar in chimeras and Sham-DCx embryos, but significantly lower in both DCx+PRL and DCx+Hyp embryos (Figure 9). No PRL was found in the plasma of DCx embryos.

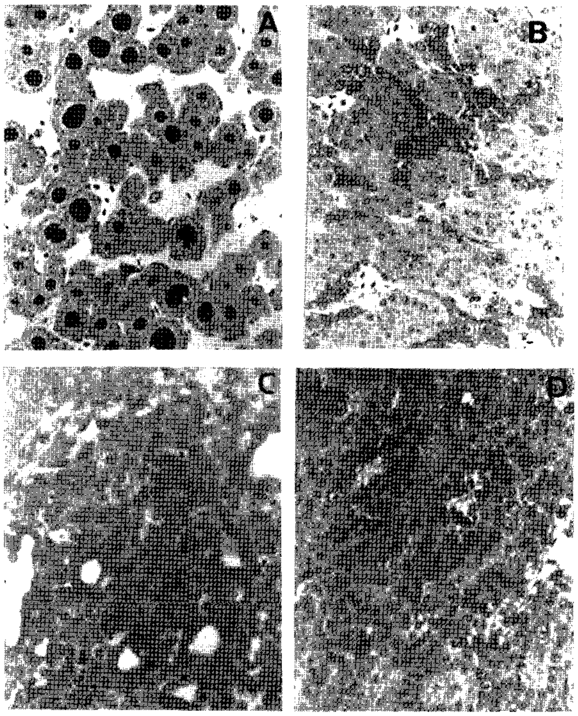

FIGURE 6 (A) Thyroid gland, (B) adrenal gland, (C) ovary, and (D) testis of a 17-day-old DCx+Hyp embryo were embedded in plastic resin for light microscopy examination. The organs resemble generally those found in control Sham-DCx embryos (Figure 8), but the histological recovery is not fully achieved. Magnification: × 175.

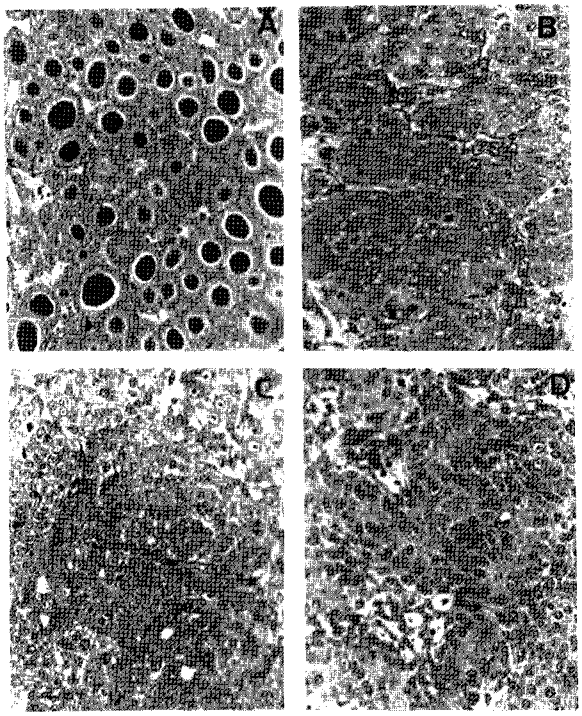

FIGURE 7 (A) Thyroid gland, (B) adrenal gland, (C) ovary, and (D) testis of a 17-day-old chimera were embedded in plastic resin for light microscopy examination. The organs exhibit the same histological features than those observed in the control Sham-DCx embryos (Figure 8). Magnification: × 175.

### DISCUSSION

We (Herradón et al., 1991; Moreno, 1994; Moreno et al., 1995) and other authors (Jankovic et al., 1978, 1981, 1982) have demonstrated profound changes in the thymus development of chicken embryos deprived of the major neuroendocrine centers by early partial decapitation (DCx embryos). In this model, the TcR$\alpha\beta$-expressing T cells seem to be specially affected, as well as the percentage of DP ($CD4^+CD8^+$), DN ($CD4^-CD8^-$), and $CD8^{lo}CD4^-$ cells, whereas $\gamma\delta$ T cells and $CD8^{hi}CD4^-$ cells show few changes. Although profound disturbance of the developing neuroendocrine system has been emphasized to be involved in these changes (Jankovic et al., 1981, 1982), precise causative agents remain unclear.

Our current results demonstrate that the treatment of DCx embryos with a single dose of 25 ng per day of recombinant chicken PRL from day 10 of incubation to the day of sacrifice induces an important, but not total, recovery of the T-cell system. In mammals, numerous data indirectly support a role for GH and PRL in the normal development of the immune system, mainly the thymus. However, in embryonic chickens, as shown by our own results, circulating levels of GH are not detected before day 17 of incubation (Harvey et al., 1979), although somatotrophic cells are present in the 12-day-old embryonic pituitary gland; it is thus impossible to assign a role for this hormone in the changes observed in the thymus of DCx embryos. In contrast, PRL appears in the chicken embryos at day 11 of incubation (Harvey et al., 1979), and increasing evidence supports its immunoregulatory influence on the adult immune system (Gala, 1991; Hooghe et al., 1993) as well as a certain relevance for thymic maturation (Hiestand et al., 1986). Thus, both Snell-Begg and Ames dwarf mice, which show important immune deficiencies,

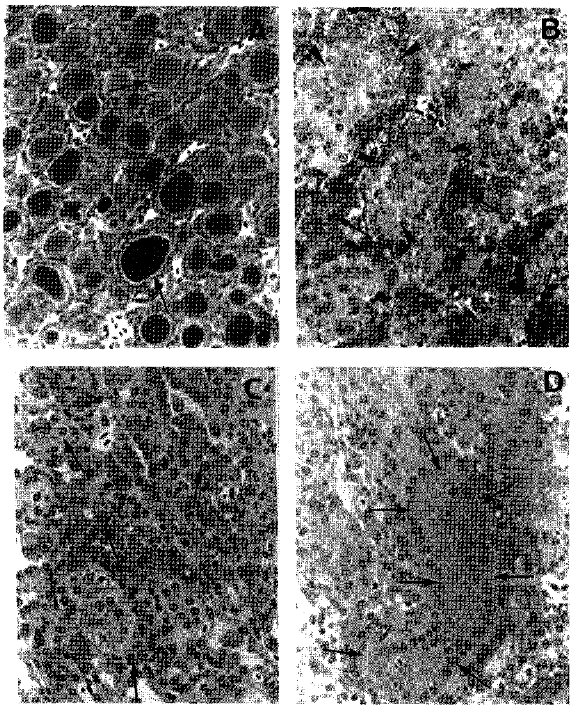

FIGURE 8 (A) Thyroid gland, (B) adrenal gland, (C) ovary, and (D) testis of a 17-day-old Sham-DCx embryo were embedded in plastic resin for light microscopy examination. Note the well-developed (A) thyroidal follicles, the chromaffin cells (arrows), and the cords of interrenal tissue (arrowheads) in the (B) adrenal gland, the groups of intersticial cells (arrows) in the (C) ovary, and the seminiferous tubules in the (D) testis (arrows). Magnification: (A) ×125; (B, C, and D) × 175.

exhibit low levels of PRL (Barkley et al., 1982), but, in contrast, dwarf children with normal values of the hormone do not present relevant immunological alterations (Gala, 1991). Moreover, Cincotta et al. (1995) reported recently that properly timed PRL administration enhances total murine thymus cell number. In agreement, our current results demonstrate that PRL supply recovers T-cell system of DCx embryos in a similar way to that observed in DCx+Hyp embryos grafted with an embryonic pituitary gland, although in this later experimental condition, the endocrine organs (i.e., thyroid, adrenal glands, and gonads) exhibit an almost normal histology. Accordingly, although other endocrine factors (see later) could be involved, PRL seems to be key factor for recovery of the T-cell system of DCx embryonic chickens.

On the other hand, even low levels of PRL seem be sufficient to bring about this recovery, confirming the previous evidence that a slight, even temporary, rise in PRL concentration has important effects on the mammalian immune system (Meltzer et al., 1983; Rovensky et al., 1991). In DCx+PRL embryos, the rapid metabolization of PRL, as previously shown in mice (Cross et al., 1992), may account for the low levels of hormone found. In addition, in chickens, unlike mammals, the hypothalamus stimulates both production and release of PRL (Kragt and Meites, 1965; El Halawani et al., 1984). Also the numbers of histologically identifiable PRL-producing cells in the grafted pituitary glands were very low (data not shown). There is, therefore, a slight increase of PRL levels in 15-day-old DCx+Hyp embryos, but the absence of hypothalamus impedes the elevation of circulating PRL, which in normal embryos occurs on days 15-17 of incubation (Harvey et al., 1979).

In the DCx embryos, there is some slowing of the maturation of the first wave of T-cell precursors, which reaches the thymus on days 6.5-8 of incubation, and complete impediment of the second wave, which colonizes the 12-14-day-old thymus (Moreno et al., 1995). This results in a gradual accumulation of the most immature T-cell subsets, including DN ($CD4^-CD8^-$) and $CD8^{lo}CD4^-$ cells, and, accordingly, decreased numbers of DP ($CD4^+CD8^+$) and mature $CD3^+TcR\alpha\beta^{hi}$ thymocytes. The recovery observed in our current results suggests, therefore, that PRL could be providing direct or indirect signals for the maturation of DN thymocytes.

In mammals, several authors have claimed a direct role for PRL in the intrathymic T-cell maturation (Pierpaoli et al., 1976; Singh and Owen, 1976; Russell et al., 1988; Gala, 1991), with both DN ($CD4^-CD8^-$) and DP ($CD4^+CD8^+$) thymocytes as the main target cells for PRL (Cross et al., 1992; Murphy et al., 1992a, 1992b). However, other immunocompetent cell types, including peripheral $CD8^+$ or $CD4^+$ cells and B lymphocytes, also respond to the hormone (Russell et al., 1988; Mukherjee et al., 1990; Gagnerault et al., 1993; Viselli and Mastro 1993). Aged rats, which accumulate DN ($CD4^-CD8^-$) cells resulting in a defect in DP thymocytes, almost entirely recover their DP ($CD4^+CD8^+$) cell compartment after implantation of GH3 cells, which produce both GH and PRL (Li et al., 1992). DW/J dwarf mice exhibit a DP thymocytes deficiency, a consequence of a slight increase of mature $CD4^+CD8^-$ cells and the migration of DP ($CD4^+CD8^+$) cells to periphery, but, principally, because of the accumulation of immature DN ($CD4^-CD8^-$) cells (Murphy et al., 1992a). Because GH administration only slightly corrects the defect, the authors conclude that the PRL plays a role in the observed alterations. This view is also supported by results that demonstrate a remarkable recovery in the number of DP ($CD4^+CD8^+$) thymocytes of dwarf mice, when the time of weaning is considerably delayed (Cross et al., 1992), indirectly suggesting that maternal PRL is the causative agent for this improvement.

In general, PRL seems to be involved in cell proliferation and maturation of immune responses (Bhat et al., 1983; Skwarlo-Sonta, 1990; Berczi et al., 1991), acting through the IL-2/IL-2R complex (Mukherjee et al., 1990; Matera et al., 1991; Viselli et al., 1991) and the expression of specific PRL receptors on different lymphoid-cell subsets (Russell

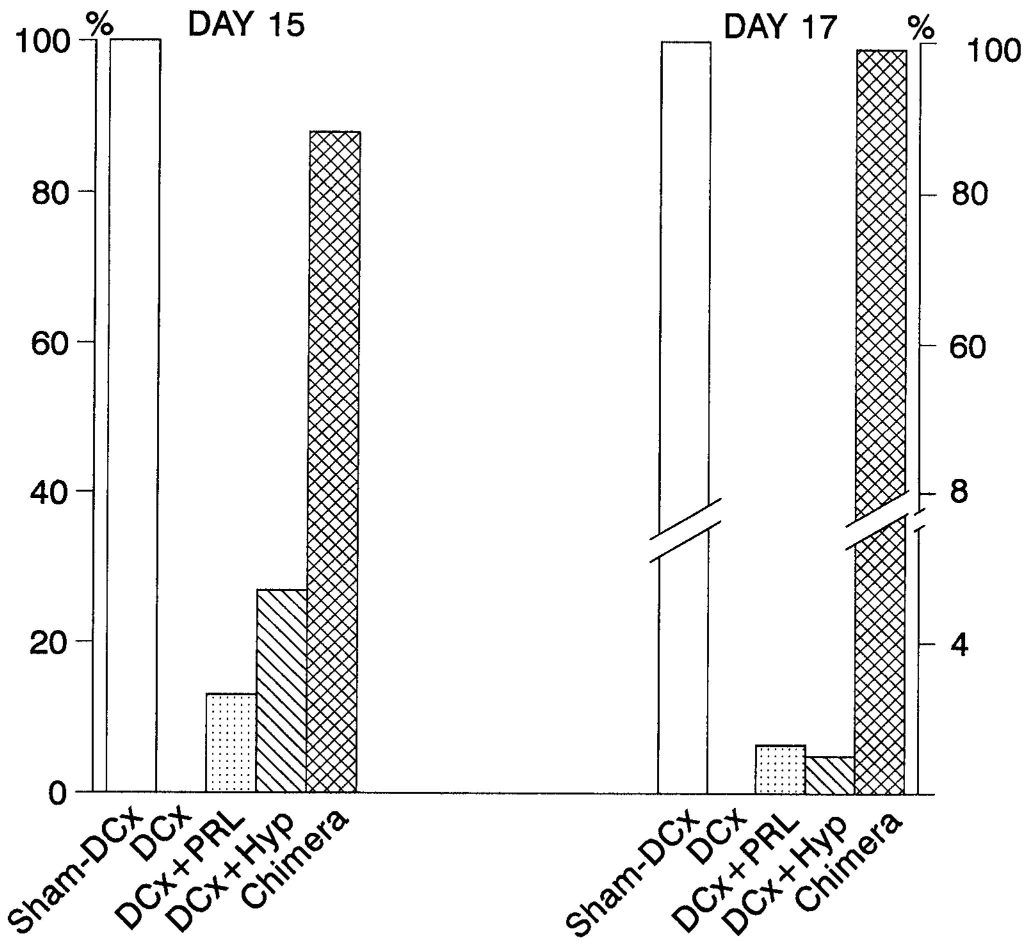

FIGURE 9 Relative levels of plasma PRL in DCx, DCx + PRL, and DCx + Hyp embryos and chimeras to those found in Sham-DCx embryos at 15 and 17 days of incubation.

et al., 1985; Bellusi et al., 1987; Pellegrini et al., 1992; Koh and Phillips, 1993; Viselli and Mastro, 1993). Although, there is not information available on the condition in chickens, indirect evidence suggests a similar situation. PRL affects the proliferative response of chicken thymocytes and splenocytes, although the cell subsets involved have not been identified (Skwarlo-Sonta, 1990). In addition, both chicken and turkey PRL induces *in vitro* proliferation of rat Nb2 cells (Soares and Proudman, 1991).

We can speculate, therefore, that PRL could mediate chicken T-cell differentiation by regulating the expression of IL-2 receptors on DN ($CD4^-CD8^-$) cells. Thus, in the absence of hormone, as occurs in DCx embryos, the number of DN ($CD4^-CD8$) IL-2R+ thymocytes decreases to stop the maturation of T-cell precursors. Why, however, does the T-cell system in the three experimental models used not totally recover, including the chick-quail chimeras?. Because, principally in DCx+Hyp and, obviously, in DCx+PRL embryos, recovery of the neuroendocrine system is incomplete, other neuroendocrine factors, apart from PRL, might be involved in the regulation of chicken T-cell differentiation. Thyroid hormones have important effects on chicken immune system (see review in Marsh and Erf, 1996) although results are frequently controversial and a target lymphoid cell for thyroxine remains to be clearly identified. Nevertheless, Glick (1984) indicated that the administration of propylthiouracyl, an inhibitor of thyroxine synthesis, induced an imbalance in the thymic cortex/medulla ratio, similar to that found in DCx embryos (Moreno et al., 1995). On the other hand, the important recovery observed in 17-day-old embryos in the three experimental models used also suggests that the length of treatment is insufficient for full recovery. Unfortunately, the drastic experimental procedure used made it impossible to obtain DCx, DCx+PRL, or DCx+Hyp embryos later than 17-18 days of incubation. Accordingly, research in progress is analyzing the recovery of 10-day-old embryonic thymus organ cultures provided with PRL, thyroxine, or both hormones, so that we can confirm or reject our hypothesis.

### MATERIALS AND METHODS

### Embryos and Surgical Procedure

Fertile eggs of White Leghorn chickens were purchased from a local supplier and hatched under standard conditions in a forced-draft incubator at 38 ± 1°C and 80% humidity. The embryo age was estimated by the duration of incubation and a minimum of four embryos of studied stages and methodological procedure were used.

At 33-38 hr (stage 10 of Hamburger and Hamilton), chicken embryos were partially decapitated (DCx embryos) according to Fugo's method (Fugo, 1940). Briefly, a window was opened in the shell and a cross-section was made through the midportion of the embryonic prosencephalon. The free anterior portion of the head was then extirpated by suction. The window was closed with adhesive tape and the egg returned to the incubator until sacrifice. Embryos 33-38 hr old, whose shells were opened and closed, served as control, sham-decapitated (Sham-DCx) animals.

For the recovery of DCx embryos, three different experimental approaches were used. A group of DCx embryos was treated by dipping on the chorioallantoid membrane a single dose/day of 25 ng of chicken prolactin (kindly provided by A. F. Parlow, Pituitary Hormones and Antisera Center, Torrance, CA) diluted in 100 $\mu$l of PBS from day 10 of incubation to the day of sacrifice (DCx+PRL embryos). In another group, the pituitary glands from 11-day-old embryonic chickens were grafted onto the chorioallantoid membrane of 11-day-old DCx embryos. A third group of animals consisted of cephalic chick-quail chimeras made by replacing the anterior midportion of the prosencephalon of a 33-38-hr chicken embryo with the same portion of a 30-33-hr quail embryo brain (chimera). The success of the different surgical procedures was achieved by routine histological sectioning either of the head or the grafted pituitary gland.

All embryos were inspected daily for viability and sacrificed on days 15 and 17 of incubation. Blood samples were taken from the chorioallantoid membrane and the plasma saved for measurement of both PRL and GH levels. Thymic lobes were aseptically removed and processed either for immunohistochemical or flow cytometrical analysis (see later). In order to evaluate the endocrinological background of different groups of studied embryos, thyroid, adrenal glands, and gonads were also fixed in Bouin's fixative and embedded in plastic resin for light microscopy examination.

### Immunohistochemistry

Thymic lobes aseptically removed were snap frozen in liquid nitrogen and stored at −80°C until use. Cryosections of 8 μm were fixed in acetone for 10 min and dried overnight. Endogenous peroxidase was blocked with 1% $H_2O_2$ in methanol. After washing in PBS, the sections were incubated for 1 hr with mAbs, CVI-His-C7 (pan-leucocytes), CT4 (anti-CD4), or CT8 (anti-CD8) (Table I) and a rabbit anti-mouse Ig conjugated to horseradish peroxidase (DAKO-Immunoglobulins, Glostrup, Denmark) diluted 1:40 in PBS for 1 hr. The peroxidase was revealed using 0.05% 3,3′ diaminobenzidine (Sigma Co., St. Louis) as chromogen and diluted in PBS plus 0.01% $H_2O_2$. Sections were counterstained with methylene blue, gradually dehydrated with graded alcohol and mounted in DePex. Negative controls were carried out on successive sections that received only the second antibody, whereas *in situ* immunostained thymic sections from 2-week-old chickens were used as positive controls. Histological sections were photographed with a Labophot (Nikon) light microscope fitted with Agfapan APX 100 film (Agfa, Leverkusen, Germany).

### Flow Cytometry

Thymic cells prepared by gently pressing through a steel mesh were suspended in PBS containing 2% FCS and 0.1% $NaN_3$ (pH 7.2). For one-color analysis, $0.5 \times 10^6$ cells were incubated with the specific mAbs listed in Table I for 30 min and, after PBS washing, with FITC-conjugated rabbit anti-mouse Ig (DAKO-Immunoglobulins, Glostrup, Denmark). For two-color analysis, one more incubation was achieved with PE-conjugated CT8 mAb. Relative immunofluorescence intensities were measured by flow cytometry with a FACScan (Becton-Dickinson, San José, CA). FACScan plus and PC-lysis softwares were used for analysis of the results.

### Radioimmunoassays

Plasma PRL and GH concentrations were measured in 75-μl aliquots by homologous double antibody RIA with chicken hormones and specific antibodies, kindly supplied by Dr. A. F. Parlow (Pituitary Hormone and Antisera Center, Harbor UCLA Medical Center, Torrance, CA). The average plasma PRL and GH values are reported in terms of chicken PRL and GH reference preparations AFP-103228B and AFP-9020C, respectively. Samples were run in a single assay to eliminate interassay variance. The intrassay coefficient of variation was 6%.

### Statistics

In the tables, each datum represents the mean value ± standard errors of percentages of positive cells of at least three different experiments. Significant differences were evaluated by Student's $t$ test and differences of $p \leq 0.1$ or $p \leq 0.05$ with control (Sham-DCx embryos) are marked.

***Acknowledgements***

We thank Drs. Max D. Cooper, C. H. Chen, O. Vainio, R. L. Boyd, and S. Jeurissen for the gift of monoclonal antibodies, Dr. A. F. Parlow for kindly providing RIA reagents, Dr. A. Esquifino for her expert assistance with RIA, and A. Cortés for help with pictures. This work was supported in part by CAICYT grant numbers PB91-0374 and PB94-0332 from the Spanish Ministry of Education and Science, FIS grant number 94-1528, and grant number 93-4758 from the Complutense University of Madrid.

***References***

Barkley M.S., Bartke A., Gross D.S. and Sinha Y.N. (1982). Prolactin status of hereditary dwarf mice. *Endocrinology*, **110**: 2088-2096.

Baroni C. (1967). Thymus peripheral lymphoid tissues and immunological responsiveness of the pituitary dwarf mouse. *Experientia*, **23**: 182-183.

Baroni C., Fabris N. and Bertoli G. (1967). Age dependency of primary immune response in hereditary pituitary dwarf and normal Snell-Bagg mouse. *Experientia*, **23**: 1059-1060.

Bellusi G., Mucciollu G., Ghe C. and Di Carlo R. (1987). Prolactin binding sites in human erythrocytes and lymphocytes. *Life Sci.*, **41**: 951-952.

Berczi I., Nagy E., De Toledo S.M., Matusik R.J., and Friesen H.G. (1991). Pituitary hormones regulate c-*myc* and DNA synthesis in lymphoid tissue. *J. Immunol.*, **146**: 2201-2206.

Berczi I., Nagy E., Kovacs K. and Horvath E. (1981). Regulation of humoral immunity in rats by pituitary hormones. *Acta Endocrinol. Copen.*, **98**: 506-513.

Bhat G., Gupta S.K. and Maiti B.R. (1983). Influence of prolactin on mitotic activity of the bursa of Fabricius in the chick. *Gen. Comp. Immunol.*, **52**: 452-455.

Cincotta A.H., Knisely T.L., Landry R.J., Miers W.R., Gutierrez P.J.A., Esperanza P. and Meier A.H. (1995). The immunoregulatory effects of prolactin in mice are time of day dependent. *Endocrinology*, **136**: 2163-2171.

Cross R.J., Bryson J.S. and Roszman T.L. (1992). Immunologic disparity in the hypopituitary dwarf mouse. *J. Immunol.*, **148**: 1347-1352.

Duquesnoy R.J. (1972). Immunodeficiency of the thymus-dependent system of the Ames dwarf mouse. *J. Immunol.*, **108**: 1578-1590.

El Halawani M.E., Silby J.L., Fehrer S.C. and Behnke E.J. (1984). Inhibitory influence of catecholamines on prolactin release in the turkey (*Meleagris gallopavo*). *Gen. Comp. Endocrinol.*, **54**: 339-341.

Esquifino A.I., Villanca M.A., Szary A., Yau J. and Bartke A. (1991). Ectopic pituitary transplants restore immunocompetence in Ames dwarf mice. *Acta Endocrinol. Copen.*, **125**: 67-72.

Fabris N., Mocchegiani E., Mariotti S., Pacini F. and Pinchera A. (1989). Thyroid-thymus interactions during development and aging. *Horm. Res.*, **31**: 85-89.

Fugo N.W. (1940). Effects of hypophysectomy in the chick embryo. *J. Exp. Zool.*, **85**: 271-297.

Gagnerault M.C., Touraine P., Savino W., Kelly P.A. and Dardenne M. (1993). Expression of prolactin receptors in murine lymphoid cells in normal and autoimmune situations. *J. Immunol.*, **150**: 5673-5681.

Gala R.R. (1991). Prolactin and growth hormone in the regulation of the immune system. *Proc. Soc. Exp. Biol. Med.*, **198**: 513-527.

Glick B. (1984). Interrelation of the avian immune and neuroendocrine systems. *J. Exp. Zool.*, **232**: 671-682.

Harvey S., Davidson T.F. and Chadwick A. (1979). Ontogeny of growth hormone and prolactin secretion in the domestic fowl (*Gallus domesticus*). *Gen. Comp. Endocrinol.*, **39**: 270-273.

Herradón P.G., Razquín B. and Zapata A.G. (1991). Effects of early partial decapitation on the ontogenic development of chicken lymphoid organs. *I. Thymus. Amer. J. Anat.*, **191**: 57-66.

Hiestand P.C., Mekler P., Nordmann R., Grieder A. and Permmongkol P. (1986). Prolactin as a modulator of lymphocyte responsiveness provides a possible mechanism of action for cyclosporine. *Proc. Natl. Acad. Sci. USA*, **83**: 2599-2603.

Hooghe R., Delhase M., Vergani P., Malur A. and Hooghe-Peters E.L. (1993). Growth hormone and prolactin are paracrine growth and differentiation factors in the haemopoietic system. *Immunol. Today*, **14**: 212-214.

Jankovic B.D., Isakovic K. and Knezevic Z. (1978). Ontogeny of the immuno-neuro-endocrine relationship. Changes in lymphoid tissues of chick embryos surgically decapitated at 33-38 hours of incubation. *Dev. Comp. Immunol.*, **2**: 479-492.

Jankovic B.D., Isakovic K. and Micic M. (1982). The thymus-hypophysis interactions in the developing chick embryo: Thymic epithelial cells in hipophysectomized embryos. In: In Vivo Immunology: Histophysiology of the Lymphoid System, Nieuwenhuis P., van der Broek A.A., and Hanna M.G., Eds. (New York: Plenum Press), pp. 343-348.

Jankovic B.D., Isakovic K., Micic M. and Knezevic Z. (1981). The embryonic lympho-neuro-endocrine relationship. *Clin. Immunol. Immunopathol.*, **18**: 108-120.

Koh C.Y. and Phillips J.T. (1993). Prolactin receptor expression by lymphoid tissues in normal and immunized rats. *Mol. Cell. Endocrinol.*, **92**: R21-R25.

Kragt C.L. and Meites J. (1965). Stimulation of pigeon pituitary prolactin release by pigeon hypothalamic extract in vitro. *Endocrinology*, **76**: 1169-1176.

Li Y.M., Brunke D.L., Dantzer R. and Kelley K.W. (1992). Pituitary epithelial cell implants reverse the accumulation of $CD4^-CD8^-$ lymphocytes in thymus glands of aged rats. *Endocrinology*, **130**: 2703-2709.

Marsh J.A. and Erf G.F. (1996). Thyroid hormones. In: The Physiology of Immunity, Marsh J.A. and Kendall M.D., Eds. (Boca Raton, FL: CRC Press), in press.

Matera L., Bellone G. and Cesano A. (1991). Prolactin and the neuroimmune network. *Adv. Neuroimmunol.*, **1**: 158-172.

Meltzer H.Y., Simonovic M. and Gudelsky G.A. (1983). Effect of yohimbine on rat prolactin secretion. *J. Pharmacol. Exp. Ther.*, **224**: 21-27.

Moreno J. (1994). Efectos de la prolactina sobre el desarrollo ontogénico del sistema T del pollo (*Gallus gallus*). Ph.D. diss., Complutense University, Madrid, Spain.

Moreno J., Vicente A., Varas A. and Zapata A.G. (1995). T-cell development in early partially decapitated chicken embryos. *Dev. Immunol.*, **4**: 211-226.

Mukherjee P., Mastro A.M. and Hymer P.S. (1990). Prolactin induction of interleukin-2 receptors on rat splenic lymphocytes. *Endocrinology*, **126**: 88-94.

Murphy W.J., Durum S.K., Anver M.R. and Longo D.L. (1992a). Immunologic and hematologic effects of neuroendocrine hormones. Studies on DW/J dwarf mice. *J. Immunol.*, **148**: 3799-3805.

Murphy W.J., Durum S.K. and Longo D.L. (1992b). Role of neuroendocrine hormones in murine T cell development. Growth hormone exerts thymopoietic effects in vivo. *J. Immunol.*, **149**: 3851-3857.

Murphy W.J., Durum S.K. and Longo D.L. (1993). Differential effects of growth hormone and prolactin on murine T cell development and function. *J. Exp. Med.*, **178**: 231-236.

Pellegrini L., Lebroun J.J., Ali S. and Kelly P.A. (1992). Expression of prolactin and its receptors in human lymphoid cells. *Mol. Endocrinol.*, **6**: 1023-1031.

Pierpaoli W., Kopp H.G. and Bianchi E. (1976). Interdependence of thymic and neuroendocrine function in ontogeny. *Clin. Exp. Immunol.*, **24**: 501-506.

Rovensky J., Vigas M., Marek J., Blazickova S., Korkakova L., Vyletelkova L. and Takac A. (1991). Evidence for immunomodulation properties of prolactin in selected in vitro and in vivo situations. *Int. J. Immunopharmac.*, **13**: 267-272.

Russell D.H., Kibler R., Matrisian L., Larson D.F., Paulos B. and Magon B.E. (1985). Prolactin receptors on human T- and B-lymphocytes: Antagonism of prolactin binding by ciclosporine. *J. Immunol.*, **134**: 3207-3213.

Russell D.H., Mills K.T., Talamantes F.J. and Bern H.A. (1988). Neonatal administration of prolactin antiserum alters the developmental pattern of T- and B-lymphocytes in the thymus and spleen of BALB/c female mice. *Proc. Natl. Acad. Sci. USA*, **85**: 7404-7407.

Singh U. and Owen J.T.T. (1976). Studies on the maturation on thymus stem cells. The effects of catecholamines, histamine and peptide hormones on the expression of T-cell alloantigens. *Eur. J. Immunol.*, **6**: 59-62.

Skwarlo-Sonta K. (1990). Mitogenic effects of prolactin on chicken lymphocytes in vitro. *Immunol. Lett.*, **24**: 171-178.

Soares M.J. and Proudman J.A. (1991). Turkey and chicken prolactins stimulate the proliferation of rat Nb2 lymphoma cells. *Proc. Soc. Exp. Biol. Med.*, **197**: 384-386.

Viselli S.M. and Mastro A.M. (1993). Prolactin receptors are found on heterogeneous subpopulations of rat splenocytes. *Endocrinology*, **132**: 571-576.

Viselli S.M., Stanek E.M., Muicherje P., Hymer W.C. and Mastro A.M. (1991). Prolactin induced mitogenesis of lymphocytes from ovariectomized rats. *Endocrinology*, **129**: 983-990.

